# Aldosterone-stimulated endothelial epithelial sodium channel (EnNaC) plays a role in cold exposure–induced hypertension in rats

**DOI:** 10.3389/fphar.2022.970812

**Published:** 2022-10-06

**Authors:** Liang-Liang Tang, Xu Yang, Shu-Qi Yu, Qi Qin, Rong Xue, Yu Sun, Han Xiao, An-Qi Shang, Jia-Qun Liu, Song-Qi Han, Chen Liang, Jie Lou, Qiu-Shi Wang, Chang-Jiang Yu, Ming-Ming Wu, Zhi-Ren Zhang

**Affiliations:** ^1^ Departments of Pharmacy and Cardiology, Harbin Medical University Cancer Hospital, Institute of Metabolic Disease, Heilongjiang Academy of Medical Science, Heilongjiang Key Laboratory for Metabolic Disorder and Cancer Related Cardiovascular Diseases, Harbin, China; ^2^ Department of Cardiology, the 1st Affiliated Hospital of Harbin Medical University, NHC Key Laboratory of Cell Transplantation, Harbin Medical University and Key Laboratories of Education Ministry for Myocardial Ischemia Mechanism and Treatment, Harbin, China

**Keywords:** ENaC, cold exposure, vascular dysfunction, hypertension, aldosterone

## Abstract

**Background:** Previous studies have demonstrated that activated endothelial epithelial sodium channel (EnNaC) impairs vasodilatation, which contributes to salt-sensitive hypertension. Here, we investigate whether mesenteric artery (MA) EnNaC is involved in cold exposure–induced hypertension (CIH) and identify the underlying mechanisms in SD rats.

**Methods:** One group of rats was housed at room temperature and served as control. Three groups of rats were kept in a 4°C cold incubator for 10 h/day; among which two groups were administrated with either benzamil (EnNaC blocker) or eplerenone (mineralocorticoid receptor antagonist, MR). Blood pressure (BP), vasodilatation, and endothelial function were measured with tail-cuff plethysmography, isometric myograph, and Total Nitric Oxide (NO) Assay kit, respectively. A cell-attached patch-clamp technique, in split-open MA, was used to determine the role of EnNaC in CIH rats. Furthermore, the plasma aldosterone levels were detected using an ELISA kit; and Western blot analysis was used to examine the relative expression levels of Sgk1 and Nedd4-2 proteins in the MA of SD rats.

**Results:** We demonstrated that cold exposure increased BP, impaired vasodilatation, and caused endothelial dysfunction in rats. The activity of EnNaC significantly increased, concomitant with an increased level of plasma aldosterone and activation of Sgk1/Nedd4-2 signaling. Importantly, CIH was inhibited by either eplerenone or benzamil. It appeared that cold-induced decrease in NO production and impairment of endothelium-dependent relaxation (EDR) were significantly ameliorated by either eplerenone or benzamil in MA of CIH rats. Moreover, treatment of MAs with aldosterone resulted in an activation of EnNaC, a reduction of NO, and an impairment of EDR, which were significantly inhibited by either eplerenone or GSK650394 (Sgk1 inhibitor) or benzamil.

**Conclusion:** Activation of EnNaC contributes to CIH; we suggest that pharmacological inhibition of the MR/Sgk1/Nedd4-2/EnNaC axis may be a potential therapeutic strategy for CIH.

## Introduction

A number of studies have demonstrated that there is a higher risk for cardiovascular diseases, as well as a higher rate of mortality in winter than that in summer (Houdas et al., 1992; Sun, 2010; Liang et al., 2017). Although multiple factors may contribute to the increased risk of cardiovascular diseases during the colder season, the increase in blood pressure (BP) due to lower temperature is considered as one of the important factors (Liu et al., 2015a; Brook, 2017). Furthermore, a large-scale retrospective study showed that there is a significant increase in mean systolic blood pressure (SBP)/diastolic blood pressure (DBP), by ∼10/4 mmHg in winter, in an Asian population (Lewington et al., 2012). Therefore, to elucidate the mechanisms by which low temperature causes elevation in BP will provide a rationale for preventing cold-induced hypertension (CIH) and its associated cardiovascular events.

Decreased production of vasodilator NO is tightly linked to endothelial dysfunction, vasoconstriction, and elevation of blood pressure. Previous studies showed that activation of EnNaC in the endothelium contributes to pathological stimuli-induced vascular endothelial dysfunction and hypertension (Jeggle et al., 2013; Wang et al., 2018; Yang et al., 2020b; Liang et al., 2021). Augmented EnNaC activity hampered the transportation of L-arginine, resulting in impaired NO generation in human umbilical vein endothelial cells (Guo et al., 2016). EnNaC inhibition has a positive effect on activation of endothelial nitric oxide synthetase (eNOS) by increasing the phosphorylation of eNOS and favoring the dimerization/coupling of eNOS (Tarjus et al., 2019). Moreover, high salt diet-elevated EnNaC activity led to impairment of vascular relaxation, which was also associated with the suppression of NO production in salt-sensitive hypertension rats (Wang et al., 2018). However, it is not known whether EnNaC plays a role in cold-induced hypertension.

Recent studies showed that DEG/ENaC acts as a thermo sensitive channel and DEG/ENaC is capable to confer warm responsiveness, with an average temperature threshold ∼32.0°C (Takagaki et al., 2020). It also reported that colder temperature, ranging from 35°C to 10°C, increased the constitutively active Na^+^ currents carried by ENaC in *Xenopus* oocytes (Askwith et al., 2001; Chraibi and Horisberger, 2003). These results suggest that ENaC may act as a cold “sensor”, even though these data were obtained from the *in vitro* data. As for EnNaC expressed in deep tissue, especially in vascular endothelial cells, where substantial temperature variations do not occur, how EnNaC located in deep tissue reacts to low temperature arouses our interest. Earlier studies showed that cold exposure–induced elevation of BP is a mineralocorticoid-induced hypertension which is sodium-dependent, even though the animals were fed with a minimum of NaCl in their diet (Sun et al., 1997; Sun et al., 2008). Other reports also suggested that cold exposure led to an increase in the aldosterone levels in both plasma and urinary tracts (Wang et al., 2005; Sun et al., 2008). Our previous data revealed that EnNaC regulated by aldosterone is essential for blood pressure control (Wang et al., 2018). Therefore, we hypothesized that aldosterone-mediated activation of EnNaC may play a role in cold-induced hypertension.

In the present study, we used cold-exposed male Sprague–Dawley (SD) rats, as the experimental model, to investigate the role of EnNaC in CIH. We demonstrated that compared with those from control SD rats, cold exposure significantly elevated EnNaC activity, reduced the endothelial NO production, impaired endothelial-dependent relaxation, and increased blood pressure in SD rats. Furthermore, inhibition of the aldosterone/Sgk1/Nedd4-2/EnNaC axis by eplerenone or benzamil significantly attenuated the cold exposure–induced increase in EnNaC activity and elevation of BP in SD rats. We suggest that the blockade of EnNaC might be a potential therapeutic strategy for treatment of CIH.

## Materials and methods

### Animals and experimental protocols

All animal care and experimental procedures were approved by the Harbin Medical University Animal Supervision Committee. All studies involving animals were reported in accordance with the ARRIVE guidelines for reporting experiments involving animals (McGrath et al., 2010). Male SD rats weighing 220–240 g (8 weeks) were purchased from the animal center of the Second Affiliated Hospital of Harbin Medical University (Harbin, China). Age-matched male SD rats were divided into six groups: 1) SD rats + room temperature (RT; 25°C); 2) SD rats + RT + benzamil; 3) SD rats + RT + eplerenone;4) SD rats + cold temperature (4°C); 5) SD rats + cold temperature + benzamil; 6) SD rats + cold temperature + eplerenone. Three group was transferred to room temperature, and other groups were exposed under 4°C conditions for 10 h/day and then maintained at room temperature (14 h/day). After cold exposure for 4 weeks, the rats were re-exposed to RT (reRT) for 1 week. Two groups of RT rats or cold-exposed rats were treated with either mineralocorticoid receptor antagonist eplerenone (100 mg/kg/day, p.o.) or with benzamil (0.7 mg/kg/day, i.p.), as previously reported (Lian et al., 2012; Zheng et al., 2016). All animals were fed normal laboratory chow, and both the diet and water were provided *ad libitum* throughout the experimental period. Heart, mesenteric artery, kidney, urine, and blood samples were collected from rats with/without cold exposure at 0, 1, 2, 3, 4 weeks, and at 5th week kept in RT (re-RT). The heart weight, body weight, and kidney weight were monitored during the span of 5 weeks.

### Blood pressure measurement

Blood pressure parameters (SBP, DBP, and MBP) and heart rate were measured weekly in conscious animals by tail-cuff plethysmography (CODA, 20310, Kent Scientific Corporation, United States), as previously reported (Liu et al., 2015b; Wang et al., 2018; Yang et al., 2020b). Briefly, the rats were secured in a rodent restrictor followed by positioned on a temperature-controlled heating pad to maintain normothermia during the testing. BP was recorded 15 times, the higher and lower recordings were discarded, and then six similar values were taken for calculating the mean BP.

### Nitric oxide measurement

The dissected mesenteric vascular beds were placed in a Petri dish containing cold PBS, and the fatty tissues and mesenteric veins were removed. Total NO production in mesenteric artery (MA) sections were determined by measuring the concentration of nitrate and nitrite, which are stable metabolites of NO, using the Total Nitric Oxide Assay Kit (S0023, Beyotime Company, China) in accordance with the manufacturer’s instructions, as described previously (Liu et al., 2015b).

### Blood and urine analysis

The blood samples were collected with or without the anticoagulant vacuum tubes for plasma preparation or serum preparation. Plasma aldosterone concentrations were measured using an ELISA kit (CEA911Ge, Cloud Clone, United States). The levels of serum K^+^ were analyzed by using a blood potassium concentration test kit (BC2775, Solarbio, China), in accordance with the manufacturer’s instructions. Urinary catecholamines containing norepinephrine, epinephrine, and dopamine were measured by using relative kits (H096, H208, and A170, Nanjing Jiancheng Bioengineering Institute, China), in accordance with the manufacturer’s instructions.

### Glomerular filtration rate

Glomerular filtration rate (GFR) was determined by calculating the creatinine clearance according to the method of Mira Farouk *et al.* (Estaphan et al., 2015). Creatinine clearance was calculated based on the following equation: creatinine clearance = *u × v/p*, where *u* means the urinary concentration of creatinine (mg/100 ml), *p* represents the plasma concentration of creatinine (mg/100 ml), and *v* indicates the urine volume (ml/min).

Both urinary creatinine and plasma creatinine were estimated by using the Creatinine Assay kit (C011-2-1, Nanjing Jiancheng Bioengineering Institute, China).

### DNA extraction and quantitative real-time PCR amplification

For MA and kidney tissues, total RNA was extracted using the TRIzol reagent and reverse-transcribed to cDNA using SuperScript II Reverse Transcriptase. The genes of α-, β-, and γ-ENaC at these tissues were quantized *via* real-time PCR as described previously (Loh et al., 2017). The primers are listed in the Supplementary Table S1.

### Myograph functional study

The vasodilatation of isolated MA rings was measured using an isometric myograph (Danish Myo Technology, Aarhus, Denmark) as described previously (Liu et al., 2015b; Wang et al., 2018; Yang et al., 2020b). Briefly, the isolated second-order MAs from SD rats were cut into 1.8–2 mm in length. The MA rings were equilibrated in PSS (composition in mM: 119 NaCl, 4.7 KCl, 2.5 CaCl_2_, 1 MgCl_2_, 25 NaHCO_3_, 1.2 KH_2_PO_4_, and 11 D-glucose) at 37°C and bubbled with a mixture of 5% CO_2_ in 95% O_2_ for 1 h in the myograph. The rings were given a resting tension of 3 mN and then allowed to equilibrate for 1 h prior to being precontracted by 10 μM phenylephrine (Phe). Endothelium-dependent and endothelium-independent relaxations were assessed by measuring dilatory responses to cumulative concentrations of acetylcholine (ACh; concentration ranging from 0.1 nM to 10 μM) and nitroglycerin (NTG; concentration ranging from 0.1 nM to 10 μM). Some MAs form rats with or without cold exposure were, respectively, incubated with 1 μM benzamil (BEN), a potent EnNaC blocker for 3 h, 10 μM eplerenone (EPL), an aldosterone receptor blocker for 3 h, or Sgk1 inhibitor 10 μM GSK650394 (GSK) for 3 h before assessing their relaxation response to ACh. For *ex vivo* experiments, the rings were incubated with 10 nM aldosterone for 3 h in the presence of or in the absence of BEN (1 μM), EPL (10 μM), or GSK (10 μM).

### *In situ* patch-clamp recording

As described previously (Liu et al., 2015b; Liang et al., 2018), *in situ* patch-clamp recording of EnNaC single-channel currents were performed using the intact vascular endothelium. The secondary segments from the superior MA were dissected as follows: the fat and connecting tissue were gently removed to avoid stretching and damaging the endothelium, and the arteries were washed with PBS to remove blood cells. The second-order MA was then cut-opened with a sharpened micropipette in order to allow the patch pipette access to the endothelial cells. To immobilize the fragments, the split-opened MAs were mounted on a plastic dish coated with L-polylysine in a recording chamber. A cell-attached configuration was used to record the EnNaC single-channel currents with an Axon Multiclamp 200B amplifier (Axon Instruments, United States) at RT. Patch pipettes were pulled from borosilicate glass with a Sutter P-97 horizontal puller, and the resistance of the pipettes ranged from 6 to 10 MΩ when filled with the pipette solution (composition in mM: 115 NaCl, 4.5 KCl, 0.1 EGTA, 5 HEPES, and 5 Na-HEPES; pH 7.2 with NaOH). The bath solutions contained (in mM) 115 NaCl, 4.5 KCl, 1 MgCl_2_, 1 CaCl_2_, 5 HEPES, and 5 Na-HEPES (pH 7.2 with NaOH). The data were acquired by the application of 0 mV to patch pipettes and were sampled at 5 kHz and low-pass filtered at 1 kHz using Clampex 10.2 software (Molecular Devices, United States). Prior to analysis, the single-channel traces were further filtered at 30 Hz and single-channel events were listed and values of the ENaC open probability (*P*_O_) were analyzed by using Clampfit 10.2 software. The I–V relationships were constructed using the single-channel amplitude, at the indicated membrane potentials (-V_pipette_) as a function of voltage, and the slope conductance was fit by linear regression using Origin 9.0 software.

### Western blot analysis

For Western blot analysis, protein samples were extracted from MAs. Protein concentrations were determined using the BCA Protein Assay Kit (APPLYGEN, Beijing, China). The proteins were separated on 10% SDS-polyacrylamide gels and transferred to nitrocellulose membranes. The membranes were blocked with 5% (w/v) BSA in Tris-buffered saline (TBS) for 1 h at RT. The membranes were, respectively, incubated with the primary antibodies against Sgk1 (1:1,000 dilution, ab59337, Abcam, United Kingdom), phospho-Sgk1 (Thr256, 1:1,000 dilution, 44-1260G, ThermoFisher, United States), Nedd4-2 (1:10,000 dilution, ab131167, Abcam, United Kingdom), phospho-Nedd4-2 (phospho S448, 1:1,000 dilution, ab168349, Abcam, United Kingdom), and GAPDH (1:10,000 dilution, ab8245, Abcam, United Kingdom) overnight at 4°C, followed by washing in 0.1% (vol/vol) Tween-20 in TBS, and incubation with secondary antibodies for another 1 h at RT. The membranes were washed with TBS-T, and the bands were quantified using the Odyssey infrared imaging system (LI-COR) and Odyssey v3.0 software.

### Primary cultured rat mesenteric artery endothelial cells

Mesenteric artery endothelial cells (MAECs) were isolated and cultured by a previously described method with modification (Wang et al., 2018). Briefly, SD rats were sacrificed by anesthesia. The mesenteric arteries were isolated and cut into small segments that were digested with collagenase IA (0.2 mg ml-1) for 50 min at 37°C. After enzyme digestion, the suspension was centrifuged at 1,200 rpm for 5 min. The sedimented cells were resuspended and plated in dishes containing DMEM/high glucose solution (100 U/mL penicillin, 100 μg/ml streptomycin, and 20% fetal bovine serum). After 40 min to allow no endothelial cells to attach to the dish, the medium was gently aspirated off and transferred to another dish. The adherent endothelial cells were cultured at 37°C under 5% CO_2_ for 3–5 days. These cells were used for experiments without further cell passage.

### Reagents

Unless otherwise noted, all reagents were purchased from Sigma-Aldrich (St. Louis). GSK650394 was purchased from Tocris (Bristol, United Kingdom).

### Statistics

Data were expressed as the mean ± SEM. Data were analyzed using the *t*-test or one-way ANOVA with repeated measurements using GraphPad Prism 5 software (GraphPad Software, Inc., San Diego, United States). A *p*-value of <0.05 was considered as statistically significant.

## Results

### Cold exposure increased blood pressure, caused endothelial dysfunction, and impaired endothelium-dependent relaxation in Sprague–Dawley rats

**FIGURE 1:**
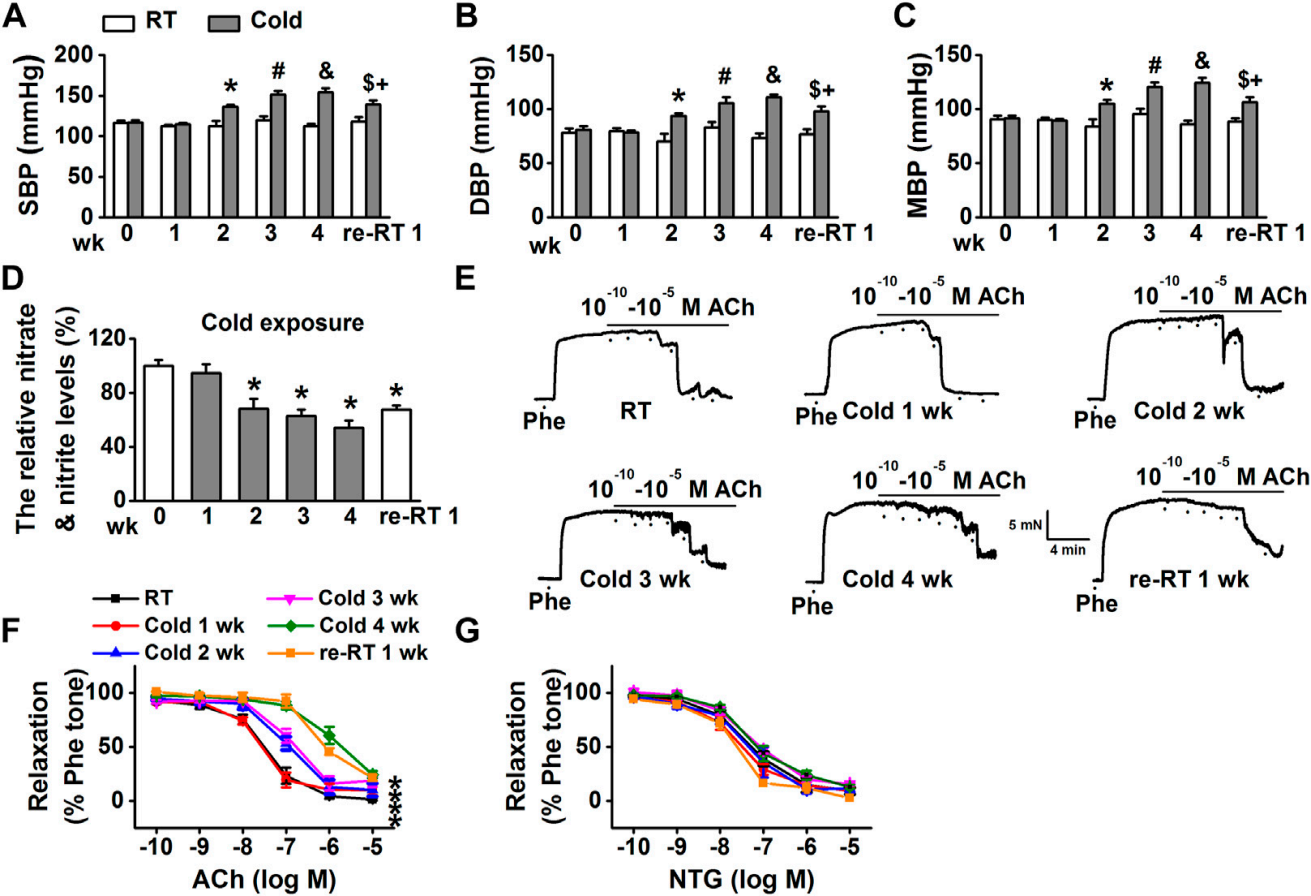
Cold exposure increased blood pressure, decreased NO production, and impaired EDR. **(A–C)** SBP, DBP, and MBP were, respectively, measured before and after cold exposure at 1, 2, 3, 4 weeks, and at 5th week kept in RT (re-RT) (*n* = 5–8 rats for each group; *p < 0.05 vs. RT at 2 weeks; #*p* < 0.05 vs. RT at 3 weeks; &*p* < 0.05 vs. RT at 4 weeks; $*p* < 0.05 vs. cold at 4 weeks; +*p* < 0.05 vs. RT at re-RT 1 week). **(D)** Concentration of nitrate and nitrite, an indirect indication of NO levels in mesenteric arteries (MAs), was, respectively, measured in dissected mesenteric vascular beds, under the control conditions and after cold exposure for 1, 2, 3, 4 weeks, and re-RT 1 week (*n* = 5 *for* each group; *p < 0.05 vs. control). **(E)** Representative traces of endothelium-dependent relaxation (EDR) of MA rings; the rings were, respectively, isolated from control rats and the cold-exposed rats at the indicated time. The MA rings were precontracted with 10 μM phenylephrine (Phe) before the application of 0.1 nM to 10 μM acetylcholine (ACh). **(F)** Summarized percent changes of EDR obtained from the experiments as shown in (E) (*n* = 5–6 *for* each group; *p < 0.05 vs. control). **(G)** Summarized percent changes of endothelium-independent relaxation in response to different doses of nitroglycerin (NTG; 0.1 nM–10 μM) in MA rings, prepared from rats with/without cold exposure for 1–4 weeks and 5th week kept in RT (row data not shown; *n* = 5–6 *for* each group).

Several studies have demonstrated that cold exposure causes vascular dysfunction and hypertension in humans and rodents (Zhu et al., 2002; Ejike et al., 2017; Zhang et al., 2019). To investigate the mechanisms underlying CIH, we established an experimental model; as shown in Figures 1A–C, compared with those of SD rats housed in RT, cold exposure significantly increased SBP, DBP, and MBP of SD rats, in a time-dependent manner. In the cold exposed group, we observed that the relative levels of nitrate and nitrite in MAs, the stable metabolites of NO, were significantly decreased 2 weeks after cold exposure in a time-dependent manner (Figure 1D). Next, we tested the effect of cold exposure on relaxation of resistance arteries. The data showed that cold exposure impaired ACh-induced EDR in the MAs of SD rats in a time-dependent manner (Figures 1E,F). However, NTG-induced endothelium-independent relaxations were not altered in the rats subjected to cold exposure (Figure 1G). Moreover, it appeared that after being exposed to cold for 4 weeks, re-exposure of these rats to RT (re-RT) for a week led to a slight, but a significant decrease in BP; the impairment of EDR and reduction of NO were not completely restored in re-RT rats compared with cold-exposed rats. Taken together, these results suggest that the SD rats under 4°C for 2 weeks exhibit hypertension due to vascular endothelial cell dysfunction and impairment of EDR, and the damage of vascular induced by cold exposure may be irreversible.

### Identification of the single-channel currents is carried by endothelial epithelial sodium channel in mesenteric artery endothelial cells

It has been well documented that renal ENaC plays an important role in regulation of blood pressure; however, we focused on investigating the role of EnNaC in CIH. First, we examined the mRNA expression profile of each EnNaC subunit in rat MA and kidney. As shown in Supplementary Figure S1A, α-, β-, and γ-EnNaCs were expressed in rat mesenteric artery tissue, but the expression abundance was much lower than that of renal ENaC. Moreover, the mRNA expression of ENaC subunits in the kidney was gradually increased with the prolongation of cold exposure (Supplementary Figures S1B–D). Next, we performed cell-attached single-channel recordings in MAECs at -V _pipette_ of 20 mV, 0 mV, −20 mV, −40 mV, and −60 mV (Supplementary Figure S1E) and constructed a plot using the single-channel amplitude as a function of voltages (-V_pipette_). The linear regression fit demonstrated that the single-channel conductance of this current is ∼5.9-pS (Supplementary Figure S1F). Furthermore, we performed the single-channel recordings in MAECs, in the presence or in the absence of benzamil in the pipette solution. The data showed that benzamil greatly blocked this current (Supplementary Figure S1G). The biophysical feature of the single-channel is consistent with those currents carried by EnNaC in endothelial cells described previously (Gu, 2008; Liu et al., 2015b; Liang et al., 2018). These results suggest that the single-channel detected in the endothelial cells of MA is carried by EnNaC.

### Cold exposure increased endothelial epithelial sodium channel activity and stimulated the aldosterone/Sgk1/Nedd4-2 signaling pathway

**FIGURE 2:**
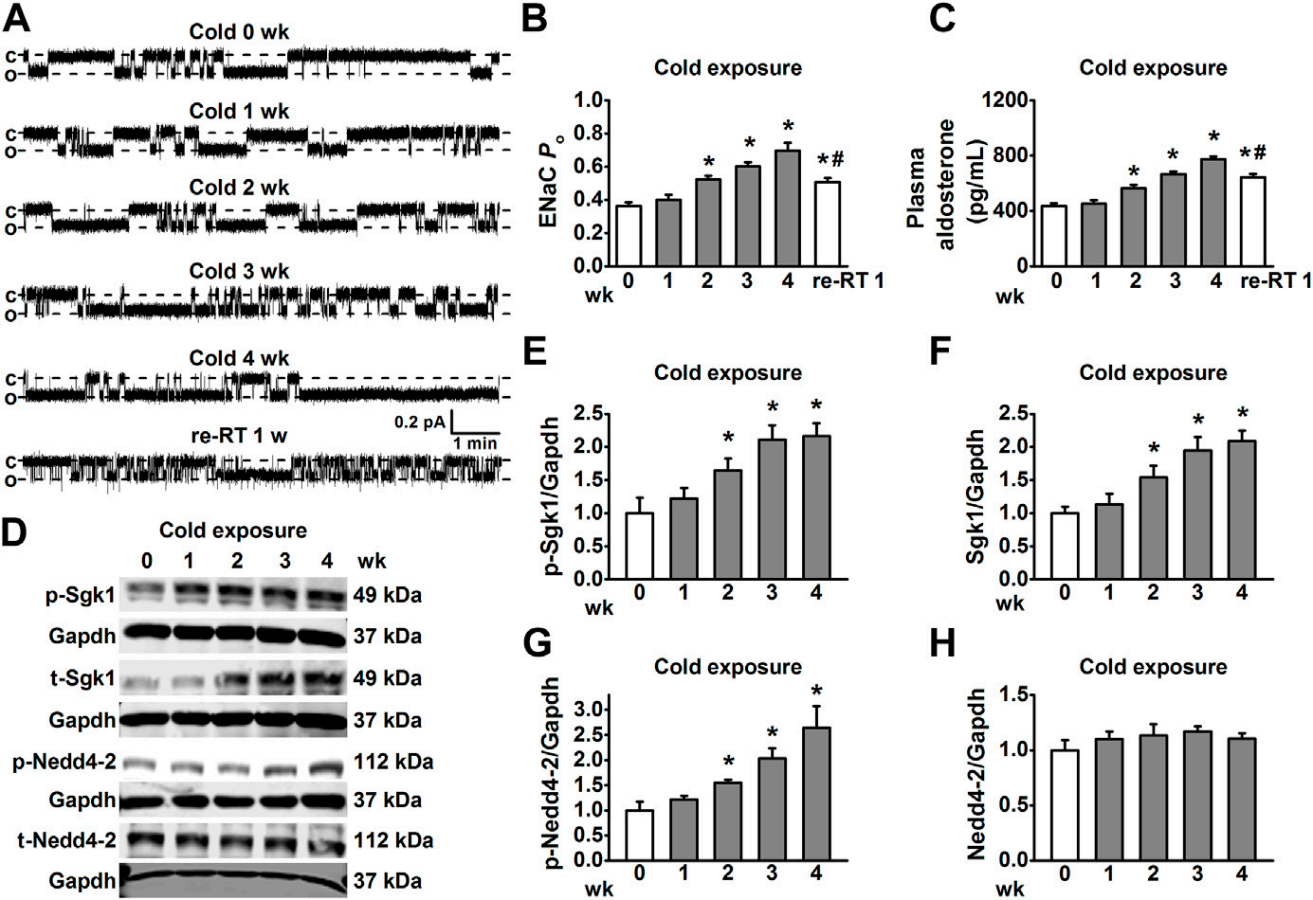
Cold exposure increased EnNaC activity in MA endothelial cells and aldosterone-mediated Sgk1/Nedd4-2 activation. **(A)** Representative EnNaC single-channel currents recorded in endothelial cells of acute split-open MAs; MAs were prepared, respectively, from control rats and the cold exposed rats (1, 2, 3, 4 weeks, and re-RT 1 week; c indicates a closed level and o represents an open level). **(B)** Demonstration of the summarized *P*_O_ values obtained from the single-channel recordings as shown in **(A)**. *P*_O_ values reflect the EnNaC activity (*n* = 5–7 *for* each group; *p < 0.05 vs control; #*p* < 0.05 vs cold exposure 4 weeks). **(C)** Demonstration of the plasma aldosterone levels in SD rats measured under the indicated conditions and time points (*n* = 5 *for* each group; *p < 0.05 vs. control; #*p* < 0.05 vs. cold exposure 4 weeks). **(D–H)** Representative Western blot images of p-Sgk1, t-Sgk1, p-Nedd4-2, and t-Nedd4-2 in MAs, isolated from rats under each indicated experimental condition and time points and the summarized expression levels of these proteins (*n* = 5 *for* each group; *p < 0.05 vs. control).

High salt-induced elevation of EnNaC activity leads to hypertension by decreasing the NO production in endothelial cells and impairment of EDR in salt-sensitive hypertension rats [9]. Therefore, *in situ* cell-attached patch-clamp experiments were performed to examine whether cold exposure may activate EnNaC in the endothelium of MAs isolated from rats. Our data showed that EnNaC activity was significantly increased 2 weeks after cold exposure in a time-dependence manner (Figures 2A,B). Usually, the thermal core of most mammas maintains relatively constant internal temperatures despite changes in ambient conditions, thus EnNaC located in thermal core organs may not respond to the cold challenge, even though it has been considered to be a cold sensor (Askwith et al., 2001; Chraibi and Horisberger, 2003). Given the fact that the CIH animal model exhibited excessive aldosterone levels (Wang et al., 2005; Sun et al., 2008), we then examined whether cold exposure could stimulate the plasma levels of aldosterone. Consistently, we found that 2 weeks after cold exposure, the levels of plasma aldosterone were significantly increased in rats, as compared with the control rats (Figure 2C). Interestingly, aldosterone concentration and ENaC activity decreased when re-placed CIH rats to RT, but the values were still higher than those before cold exposure. Therefore, we suggest that aldosterone may mediate the activation of EnNaC in CIH rats.

We further examined whether the plasma aldosterone levels and EnNaC activity could be altered by an acute cold exposure (10 h). The results showed that after 10 h of cold challenge, the plasma aldosterone levels and EnNaC activity were virtually the same as the control (Supplementary Figure S2). These results suggest that alteration of aldosterone levels may be the initiating event for activating EnNaC in CIH.

Since the Sgk1/Nedd4-2 signal pathway is the first link between the aldosterone-induced sodium transport [25], we then examined whether cold-induced increase in the levels of aldosterone may stimulate the EnNaC *via* Sgk1/Nedd4-2 signal pathway. As shown in Figures 2D–F, 2 weeks after cold exposure, both the phosphorylated and total Sgk1 levels were increased in MAs. Not surprisingly, the expression levels of phosphorylated Nedd4-2 were also significantly increased in MAs 2 weeks post cold exposure (Figure 2G), without affecting total Nedd4-2 expression throughout the cold exposure period (Figure 2H). These results together suggest that cold exposure led to excessive EnNaC activation through the aldosterone/Sgk1/Nedd4-2 axis in CIH rats.

### Eplerenone or benzamil alleviated cold exposure–induced hypertension

**FIGURE 3:**
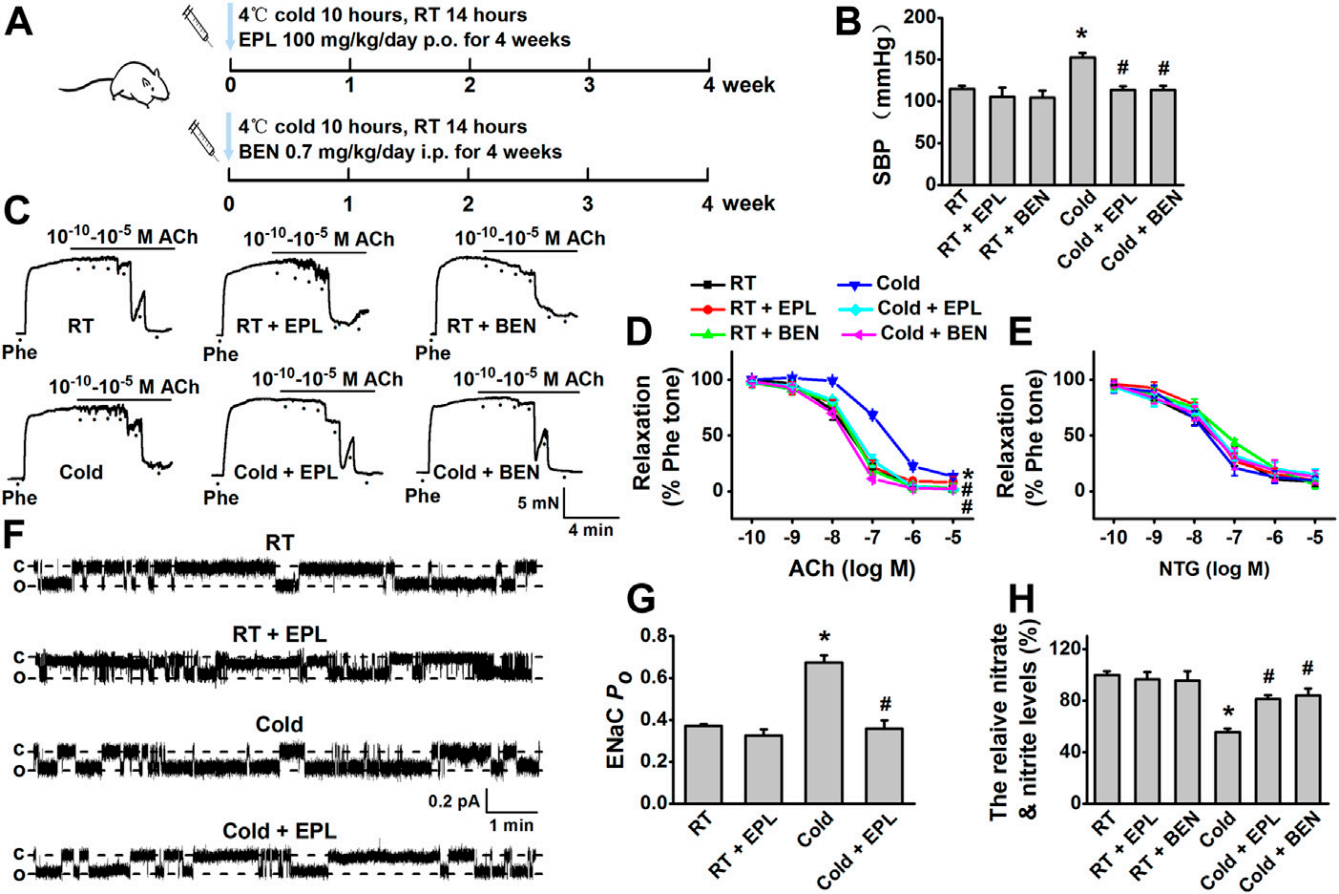
Cold exposure induced elevation of blood pressure and impairment of EDR were hindered by either eplerenone or benzamil. **(A)** Rats were kept under the 4°C/RT condition at a 10 h/14 h cycle and received the dosage of 100 mg/kg/day eplerenone (EPL, MR antagonist) or 0.7 mg/kg/day benzamil (BEN, EnNaC blocker) for 0–4 weeks. **(B)** CIH was reversed by pharmacological inhibition of aldosterone/MR or EnNaC (*n* = 5–8 *for* each group; *p < 0.05 vs. control; #*p* < 0.05 vs. cold exposed group). **(C)** Representative traces of EDR of MA rings; the rings were, respectively, isolated from the rats exposed to room temperature (RT), RT application of EPL, or BEN for 4 weeks, cold exposure, or cold exposure application of EPL or BEN for 4 weeks; the MA rings were precontracted with 10 μM Phe before the application of 0.1 nM–10 μM ACh. **(D)** Summarized percent changes of EDR obtained from the experiments as shown in **(C)** (*n* = 5–6 *for* each group; *p < 0.05 vs. control; #*p* < 0.05 vs. cold exposed group). **(E)** Summarized percent changes of endothelium-independent relaxation in response to different doses of NTG (0.1 nM–10 μM) in MA rings from each indicated group (row data not shown; *n* = 5–6 *for* each group). **(F)** Representative EnNaC single-channel currents recorded in MA endothelial cells of acute split-open MAs; MAs were, respectively, prepared from RT rats (top trace), the rats with RT for 4 weeks in the presence of EPL (the second trace), the rats with cold exposure for 4 weeks in the absence of (the third traced) or presence of EPL (bottom trace) (c indicates a closed level and o represents an open level). **(G)** Demonstration of the summarized *P*_O_ values, obtained from the single-channel recordings as shown in **(F)** (*n* = 5–7 *for* each group; *p < 0.05 vs. control; #*p* < 0.05 vs. cold exposed group). **(H)** Cold exposure (4 weeks) decreased the concentration of nitrate and nitrite in rat MAs and cold-induced reduction of nitrate and nitrite was reversed by either EPL or BEN (*n* = 5 *for* each group; *p < 0.05 vs. control; #*p* < 0.05 vs. cold exposed group).

To further confirm whether aldosterone-dependent EnNaC activation is involved in CIH, eplerenone, a MR antagonist, and benzamil, a potent EnNaC blocker, were, respectively, used to test their effects on blood pressure and vascular function during cold exposure. Our data showed that administration of eplerenone or benzamil diminished the effects of cold exposure–induced hypertension and impairment of EDR in rats (Figures 3B–D), without affecting endothelium-independent relaxations in response to NTG (Figure 3E). The data, generated from the *in situ* cell-attached patch-clamp experiments in the endothelial cells of MAs, showed that the cold-induced significant increase in EnNaC activity was reversed by the application of eplerenone (Figures 3F,G). Moreover, the cold-induced decrease in vascular NO production was also significantly increased by the application of eplerenone or benzamil (Figure 3H). Collectively, these findings suggest that aldosterone/MR interaction is the upstream signaling for activating EnNaC and that the application of MR antagonist or a direct blockade of EnNaC attenuates cold-induced impairment of EDR and enhanced blood pressure.

### Sgk1/endothelial epithelial sodium channel contributed to the detrimental effect of cold exposure or aldosterone on vasodilatation dysfunction

**FIGURE 4:**
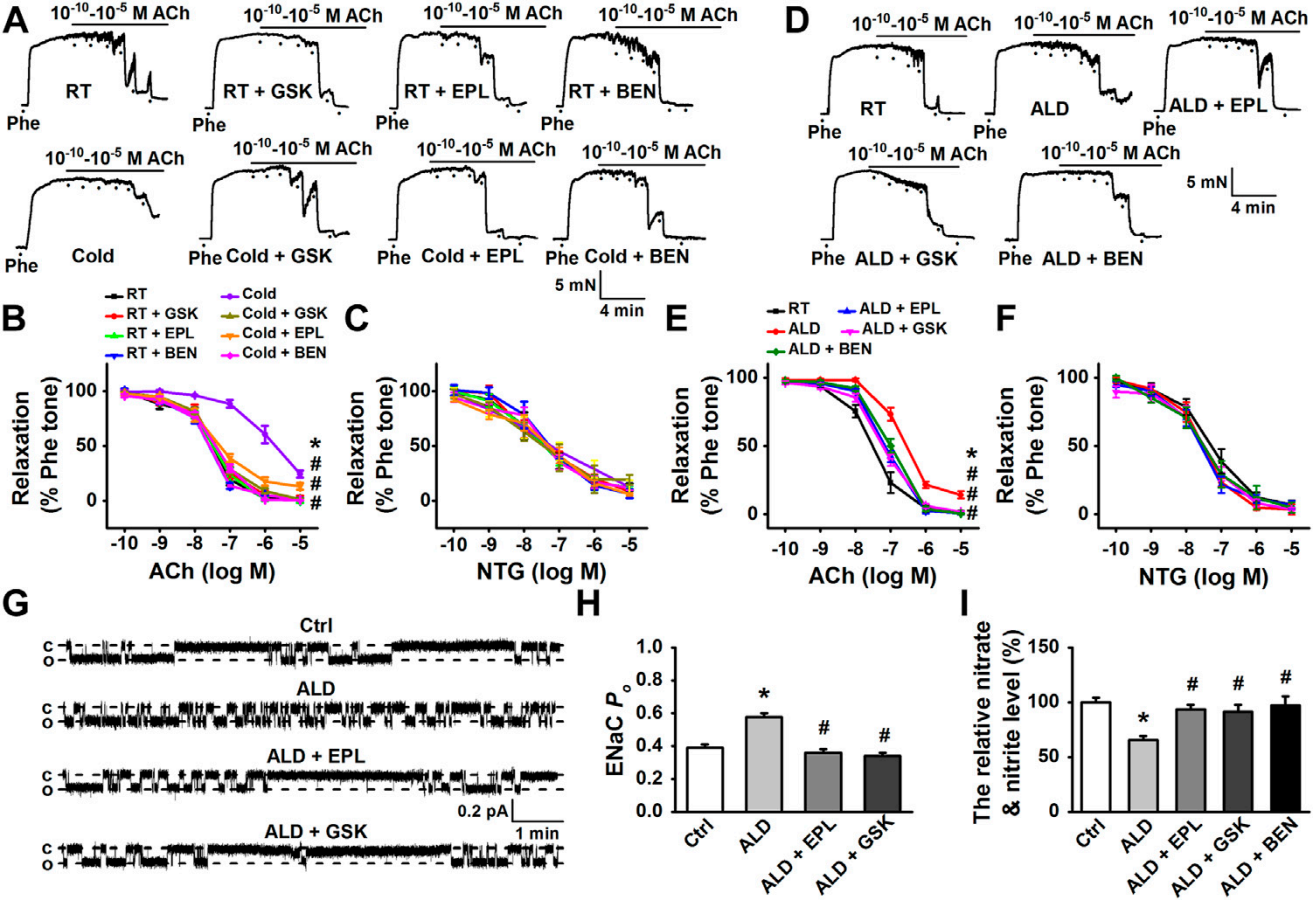
Cold- or aldosterone-induced impairment of EDR in MAs was attenuated by pharmacological inhibition of aldosterone/MR and Sgk1, or by the blockade of EnNaC. **(A)** Representative traces of EDR in MA rings; the rings were, respectively, isolated from control rats and the rats exposed to cold for 4 weeks. The rings were, respectively, treated by 10 μM eplerenone (EPL), 10 μM GSK650394(GSK), or 1 μM benzamil (BEN) for 3 h, followed by precontracting with 10 μM Phe before application of 0.1 nM–10 μM ACh. **(B)** Summarized percent changes of EDR obtained from the experiments as shown in **(A)** (*n* = 5–6 *for* each group; *p < 0.05 vs. control group; #*p* < 0.05 vs. cold exposed group). **(C)** Summarized percent changes of endothelium-independent relaxation in response to different doses of NTG (0.1 nM–10 μM) in MA rings from each indicated group (row data not shown; *n* = 5–6 *for* each group). **(D)** Representative traces of EDR in MA rings, respectively, generated from control MA, MA treated either 10 nM aldosterone (ALD), ALD plus 10 μM EPL (ALD + EPL), ALD plus 10 μM GSK (ALD + GSK), or ALD plus 1 μM BEN (ALD + BEN) for 3 h. **(E)** Summarized percent changes of EDR obtained from the experiments as shown in **(D)** (*n* = 6 *for* each group; *p < 0.05 vs. control; #*p* < 0.05 vs. ALD group). **(F)** Summarized percent changes of endothelium-independent relaxation in response to different doses of NTG (0.1 nM–10 μM) in MA rings from each indicated group (row data not shown; *n* = 6 *for* each group). **(G)** Representative EnNaC single-channel currents recorded in endothelial cells of acute split-open MAs; MAs were pretreated without (top trace) or with ALD for 3 h (the second trace) and ALD plus EPL (the third trace) and ALD plus GSK (bottom trace) (c indicates a closed level and o represents an open level). **(H)** Demonstration of the summarized *P*_O_ values, obtained from the single-channel recordings as shown in **(G)** (*n* = 7 *for* each group; *p < 0.05 vs. control; #*p* < 0.05 vs. ALD group). **(I)** ALD (10 nM) decreased the concentration of nitrate and nitrite in rat MAs and ALD-induced reduction of nitrate and nitrite was reversed by either EPL or GSK or BEN (*n* = 5 *for* each group; *p < 0.05 vs. control; #*p* < 0.05 vs. ALD group).

To confirm whether Sgk1, the downstream signaling of aldosterone contributes to cold exposure–induced dysfunction of vasodilatation, the artery rings isolated from CIH rats (cold exposure for 4 weeks) were, respectively, preincubated with 10 μM eplerenone, a MR antagonist, 10 μM GSK650394, a Sgk1 inhibitor, or 1 μM benzamil for 3 h before measuring ACh-induced relaxation. Our results showed that the cold-induced impairment of EDR was significantly improved by the application of eplerenone, GSK650394, or benzamil (Figures 4A,B), with no effects on endothelium-independent relaxations in response to NTG (Figure 4C). These findings suggest that the aldosterone-Sgk1-EnNaC pathway contributes to the cold exposure–induced impairment of vasodilatation in MAs.

In light of our finding that aldosterone was elevated in response to prolonged cold exposure, we analyzed its effect on MA vasodilatation in the *ex vivo* model, where the MA rings were pretreated with 10 nM exogenous aldosterone for 3 h. The data showed that exogenous aldosterone significantly impaired the EDR of MAs (Figures 4D,E). Consistently, pre-incubation MA rings with MR antagonist eplerenone (10 μM), GSK650394 (10 μM), or benzamil (1 μM) prevented aldosterone-induced impairment of EDR (Figures 4D,E), without affecting endothelium-independent relaxations (Figure 4F).

We further examined the effects of exogenous aldosterone on the endothelial EnNaC activity and NO production and evaluated whether the MR-Sgk1-EnNaC axis is responsible for these changes. As shown in Figures 4G–I, 10 nM aldosterone significantly increased EnNaC activity and decreased NO production. The exogenous aldosterone-induced activation of EnNaC was significantly attenuated by either pretreatment with eplerenone (10 μM) or GSK650394 (10 μM). Not surprisingly, eplerenone (10 μM), GSK650394 (10 μM), or benzamil (1 μM) restored the reduction of NO levels induced by aldosterone.

Taken together, these results suggest that cold exposure–induced dysfunction of EDR and hypertension in rats are most likely *via* aldosterone-mediated, Sgk1-dependent increase in EnNaC expression and activation of EnNaC.

### Cold exposure activated the sympathetic nervous system and reduced the glomerular filtration rate

Catecholamines are important in regulation of blood pressure; we then measured the levels of urinary catecholamines in cold-exposed rats to test whether cold may stimulate catecholamines. As shown in Supplementary Table S2, cold exposure for 4 weeks did not alter the levels of epinephrine, but increased norepinephrine and decreased dopamine with prolonged cold exposure. These data suggest that sympathetic system excitation may contribute, at least in part, to CIH.

We further examined whether cold exposure affects rat GFR. We calculated creatinine clearance to estimate GFR. As shown in Supplementary Table S2, GFR decreased in CIH rats, but had a partial recovery when transferred CIH rats to RT for 1 week. Subsequently, we also found that cold exposure did not alter the blood levels of potassium, heart–body weight ratio, kidney–body weight, and heart rate (Supplementary Table S2).

## Discussion

It appeared that the complex pathophysiological processes, including the activated sympathetic nervous system and reduced GFR, are involved in the rat CIH model. However, we found that 1) cold exposure significantly activates EnNaC in rat MA endothelial cells *via* elevating plasma aldosterone levels and stimulating its downstream regulator; 2) activated EnNaC contributes to cold exposure–induced vascular dysfunction and hypertension; 3) CIH could be ameliorated by pharmacological inhibition of EnNaC, aldosterone/MR, and Sgk1/Nedd4-2 signaling.

The biophysical features of endothelial EnNaC single-channel current, recorded in split-open MAs and in MAECs shown in this study, are comparable to those described previously in the rat or mouse endothelial cells (Liu et al., 2015b; Niu et al., 2021). Indeed, EnNaC plays a role in salt overload-, homocysteinmia-, and high-fat diet-induced vascular dysfunction (Yang et al., 2020b; Liang et al., 2021; Niu et al., 2021). In addition, other reports showed that activation of EnNaC is responsible for endothelial stiffness and vascular remodeling (Guo et al., 2016). These findings provide a clue that EnNaC may be a potential candidate for preventing cardiovascular disease, due to its widespread expression in the vascular system. Previous studies documented that cold temperature resulted in endothelial dysfunction in humans and animals; the main reason has been referred as a loss of NO bioavailability due to reduced eNOS (Ejike et al., 2017; Zhang et al., 2019). However, how low temperature promotes endothelial dysfunction, in other words, impaired NO pathway, is still not fully understood. In our CIH rat model, we found that the EDR of MAs was significantly impaired, while endothelium-independent relaxation was not affected. More importantly, the activation of EnNaC in MAs was gradually increased 2 weeks after cold exposure, with a concomitant decreased NO levels. These results strongly suggest that EnNaC is involved in CIH. This notion was further supported by the data, where administration of rats with benzamil, a potent ENaC blocker, effectively attenuated cold-induced reduction of NO levels, impairment of EDR, and elevation of BP.

Interestingly, ENaC may act as a thermo sensitivity channel (Takagaki et al., 2020) and colder temperature actives ENaC-mediated Na^+^ current in a heterologous expression system (Askwith et al., 2001; Chraibi and Horisberger, 2003). It should be noted that we examined endothelial EnNaC activity of MA, and its anatomical position is considered as “deep tissue” (interior). Thus, the exterior alteration of temperature may have the modest effect on EnNaC expressed in MA endothelial. The underlying mechanism of regulating “interior” EnNaC by cold exposure may be different from what has been seen in *Xenopus* oocytes, where lower “exterior” temperature increased EnNaC-mediated Na^+^ currents (Askwith et al., 2001). Therefore, we reasoned that cold-induced hypothermia may not directly affect the action of EnNaC in MAs.

Previous studies have shown that aldosterone is overproduced in a variety of experimental models of hypertension, including CIH (Sun et al., 1997; Wang et al., 2005) and that aldosterone is known to be a primary ENaC activator (Yang et al., 2020a). Thus, we favored the notion that the “interior” EnNaC activity might be stimulated by aldosterone rather than cold-induced hypothermia. Consistently, our data demonstrated that concomitant with the increased blood pressure, the levels of plasma aldosterone were also significantly increased by cold exposure in rats. It has been shown that the temperature and exposure time, the cold medium (air or water), and the species may lead to different outcomes, with respective to aldosterone levels (Paakkonen and Leppaluoto, 2002). Our data suggested that neither the plasma aldosterone levels nor EnNaC activity was altered 10 h post cold exposure, suggesting that acute exposure to cold does not affect aldosterone-mediated EnNaC.

As we know, Sgk1 has been identified as a primary aldosterone-induced gene in renal epithelia, which stimulates ENaC through Nedd4-2, the principal ENaC inhibitory accessory protein (Thomas et al., 2011; Valinsky et al., 2018). This is one of the main stimulatory actions of aldosterone on ENaC in the kidneys. In the present study, we proposed that endothelial EnNaC may be also regulated by the aldosterone/Sgk1/Nedd4-2 axis, because high concentration plasma aldosterone was accompanied by activated Sgk1 and Nedd4-2, as well as its-dependent EnNaC abnormal stimulation in CIH rat MAs. This notion was further supported by the results generated by the *ex vivo* experiments, where the effects, brought about by exogenous aldosterone on EnNaC, EDR, and NO production, were greatly blunted by either aldosterone/MR antagonist or Sgk1 inhibitor. These results revealed a mechanism that aldosterone-mediated increase in the expression and activation of EnNaC *via* the Sgk1/Nedd4-2 pathway ultimately leads to impairment of vasodilatation and is one of the important elements in CIH. However, we did not observe the multiple opening EnNaC channels as characterized in renal epithelial cells, even under the condition of high concentration of aldosterone, which could be due to the lower density of EnNaC expressed at the plasma membrane of MAECs, the loss of stimulatory factors, and the presence of intracellular molecules that inhibit EnNaC in intact cells (Wang et al., 2009; Paudel et al., 2022; Zhang et al., 2022).

It was reported that aldosterone-induced endothelial dysfunction and vascular remodeling are MR-dependent (Chen et al., 2019; Ferreira et al., 2021) and that aldosterone-induced aortic endothelium stiffness and impairment of EDR may associate with augmented activation of aortic EnNaC (Jia et al., 2018). Moreover, cell-specific endothelium MR deficiency prevented EnNaC-mediated western diet–induced endothelium stiffness and dysfunction of aortic relaxation in female mice (Jia et al., 2016). In addition to these previous findings, our data are consistent with the notion that interaction of aldosterone with MR plays a major role in CIH, *via* activating EnNaC. Moreover, we also observed that the expression of ENaC mRNA in the renal cortex increased in response to prolonged cold exposure, suggesting that aldosterone-stimulated ENaC activation in the distal nephron may also contribute to CIH. Thus, the protective effect of eplerenone against CIH may be beyond EnNaC and may inhibit MR-mediated renal ENaC activation as well. In this study, we did not distinguish the predominant influence between renal ENaC and vascular EnNaC in CIH, albeit the blockade of MR markedly inhibits EnNaC activity and reverses the impairment of artery relaxation in CIH rats. In addition, we found that prolonged cold exposure–induced vascular dysfunction is irreversible. The decrease in BP of CIH rats was accompanied by a moderate recovery of GFR when re-placed the rat into RT for a week. These findings, therefore, provide additional evidence that the impairment of vasodilatation was not the only factor for onset and development of CIH.

A number of evidence showed that sympathetic excitation contributes to the onset of CIH. Our results suggest that CIH rats possess high levels of norepinephrine, the critical regulator for increasing heart rate; however, the heart rate was not increased in our experimental model. Interestingly, we found that cold exposure led to a decreased release of dopamine. A previous study reported that cold exposure can significantly attenuate the number of dopamine neurons firing and protect from a pronounced increase in dopamine neuron population activity (Moore et al., 2001). These effects may induce feedback regulation, ultimately causing the lower level of dopamine. More importantly, lower dopamine levels may affect sodium transportation in the kidney (Iimura and Shimamoto, 1993), which may contribute to increased Na^+^–water retention, and thereby leading to CIH.

Overall, it appeared that the mechanisms of CIH are rather complex, involving multiple organs, including dysfunction of resistant arteries, sympathetic nervous system, hormones, and kidneys. Nevertheless, we provide the novel evidence that vascular EnNaC plays a role in aldosterone-mediated CIH, beyond a well-accepted mechanism that aldosterone is involved in excess sodium–water retention and shift in fluid balance. We suggest that aldosterone-dependent activation of EnNaC contributes to CIH and that the blockade of EnNaC or inhibiting aldosterone/MR signaling may be an effective approach to prevent cold exposure–induced vascular dysfunction and CIH.

## Data Availability

The original contributions presented in the study are included in the article/Supplementary Material; further inquiries can be directed to the corresponding author.

## Supplementary material

Supplementary Figure S1
**(A)** Relative mRNA expression profiles of α-, β-, γ-EnNaC subunits in MA and kidney were analyzed by real-time PCR and normalized to GAPDH. For each subunit, the abundance of the ENaC in renal cortex was set to 1 and used to determine the relative level and the significance of the other samples (n = −5 rats for each group; **p* < 0.05 vs. α-ENaC in renal cortex; #*p* < 0.05 vs. β-ENaC in renal cortex; &*p* < 0.05 vs. γ-ENaC in renal cortex). **(B–D)** α-, β-, γ-ENaC subunits mRNA in kidney were respectively measured after cold exposure for 0-4 weeks (n = 5 rats for each group; &*p* < 0.05 vs. cold at 0 week). **(E)** Representative single-channel currents recorded at membrane potential ranging from 20 to −60 mV in MAECs. **(F)** Currents corresponding to the voltage from cell-attached recording were plotted. The points were fitted by a linear line, slope conductance 5.9 ± 0.4-pS (n = 5 for each point). **(G)** The representative traces demonstrating the EnNaC single-channel currents recorded in the absence (the top trace) or in the presence of benzamil (the bottom trace).

Supplementary Figure S2
**(A)** Demonstrating the plasma aldosterone levels in control or cold exposure for 10 hours rats (n = 5 for each group). **(B)** Representative EnNaC single-channel currents recorded in MA isolated from control or cold exposure for 10 hours rats. **(C)** Summarized PO value obtained from the single-channel recordings as shown in **(B)**, (n = 5 for each group).

## Abbreviations

ACh, acetylcholine; ALD, aldosterone; BEN, benzamil; CIH, cold-induced hypertension; DBP, diastolic blood pressure; EDR, endothelium-dependent relaxation; EnNaC, endothelial epithelial sodium channel; EPL, eplerenone; MA, mesenteric artery; MBP, mean blood pressure; Nedd4-2, neural precursor cell expressed developmentally downregulated gene 4-2; NTG, nitroglycerin; Phe, phenylephrine; RT, room temperature; SBP, systolic blood pressure; Sgk1, serum and glucocorticoid-regulated kinase 1
