# Supplementary material for: Aldosterone-stimulated endothelial epithelial sodium channel (EnNaC) plays a role in cold exposure–induced hypertension in rats
Source: Front Pharmacol. 2022 Oct 6;13:970812. doi: 10.3389/fphar.2022.970812 (PMC9582121; doi:10.3389/fphar.2022.970812)
Supplement: Supplementary file 1 [file Table1.PDF]

Table 1

Sequences (5’-3’) of primers used.

Gene	Forward primer	Reverse primer
α-ENaC	CCTAAGCCCAAGGGAGTTGA	ACACTACAAGGCTTCCGACA
β-ENaC	TGGACATTGGTCAGGAGGAC	AGCAGCACCCCAATAGAAGT
γ-ENaC	TGAGGCTTCCGAGAAATGGT	AATACTGTTGGCTGGGCTCT
GAPDH	CAAGTTCAACGGCACAGTCA	CCCCATTTGATGTTAGCGGG
