# Supplementary material for: Aldosterone-stimulated endothelial epithelial sodium channel (EnNaC) plays a role in cold exposure–induced hypertension in rats
Source: Front Pharmacol. 2022 Oct 6;13:970812. doi: 10.3389/fphar.2022.970812 (PMC9582121; doi:10.3389/fphar.2022.970812)
Supplement: Supplementary file 2 [file Table2.PDF]

Table 2

Comparison between mean ± SEM of heart rate, heart-body weight ratio, kidney-body weight ratio, GFR, catecholamines and serum potassium levels in all studied groups.

Parameter	Cold 0 wk	Cold 1 wk	Cold 2 wk	Cold 3 wk	Cold 4 wk	Re-exposed RT 1 wk
Heart rate (beat/min)	387.92 ± 3.3	389.28 ± 22.48	381.44 ± 12.32	350.24 ± 10.51	358.32 ± 18.91	359.36 ± 19.90
Heart - Body weight (mg/g)	4.47 ± 0.25	4.86 ± 0.16	4.37 ± 0.15	4.64 ± 0.09	4.60 ± 0.14	4.20 ± 0.14
Kidney - Body weight (mg/g)	9.83 ± 0.16	10.94 ± 0.53	11.76 ± 0.44	11.55 ± 0.44	10.62 ± 0.44	10.20 ± 0.57
GFR (mL/min)	1.17 ± 0.07	1.00 ± 0.03	0.65 ± 0.02 *	0.62 ± 0.07 *	0.56 ± 0.05 *	0.87 ± 0.01 *#
Epinephrine (nmol/L)	3046.65 ± 550.56	3049.46 ± 144.96	3225.03 ± 213.04	3043.28 ± 233.65	3611.59 ± 433.76	3078.33 ± 567.81
Norepinephrine (ng/L)	22.06 ± 2.45	30.75 ± 2.48	46.84 ± 2.18 *	48.84 ± 4.18 *	53.95 ± 2.34 *	36.40 ± 3.58 *#
Dopamine (ng/mL)	1182.81 ± 23.71	1100.89 ± 37.7	908.65 ± 29.34*	880.08 ± 29.14 *	822.12 ± 27.75 *	1019.48 ± 36.53 *#
Serum potassium (µmol/mL)	7.06 ± 0.28	7.56 ± 1.11	6.16 ± 0.33	6.73 ± 0.32	6.62 ± 0.33	6.85 ± 0.39

Values are represented as mean ± SEM. n = 4-5. *Statistically significant compared to corresponding value in cold 0 wk; # Statistically significant compared to corresponding value in cold 4 wk.
