# Supplementary material for: Aldosterone-stimulated endothelial epithelial sodium channel (EnNaC) plays a role in cold exposure–induced hypertension in rats
Source: Front Pharmacol. 2022 Oct 6;13:970812. doi: 10.3389/fphar.2022.970812 (PMC9582121; doi:10.3389/fphar.2022.970812)
Supplement: Supplementary file 5 [file DataSheet1.PDF]

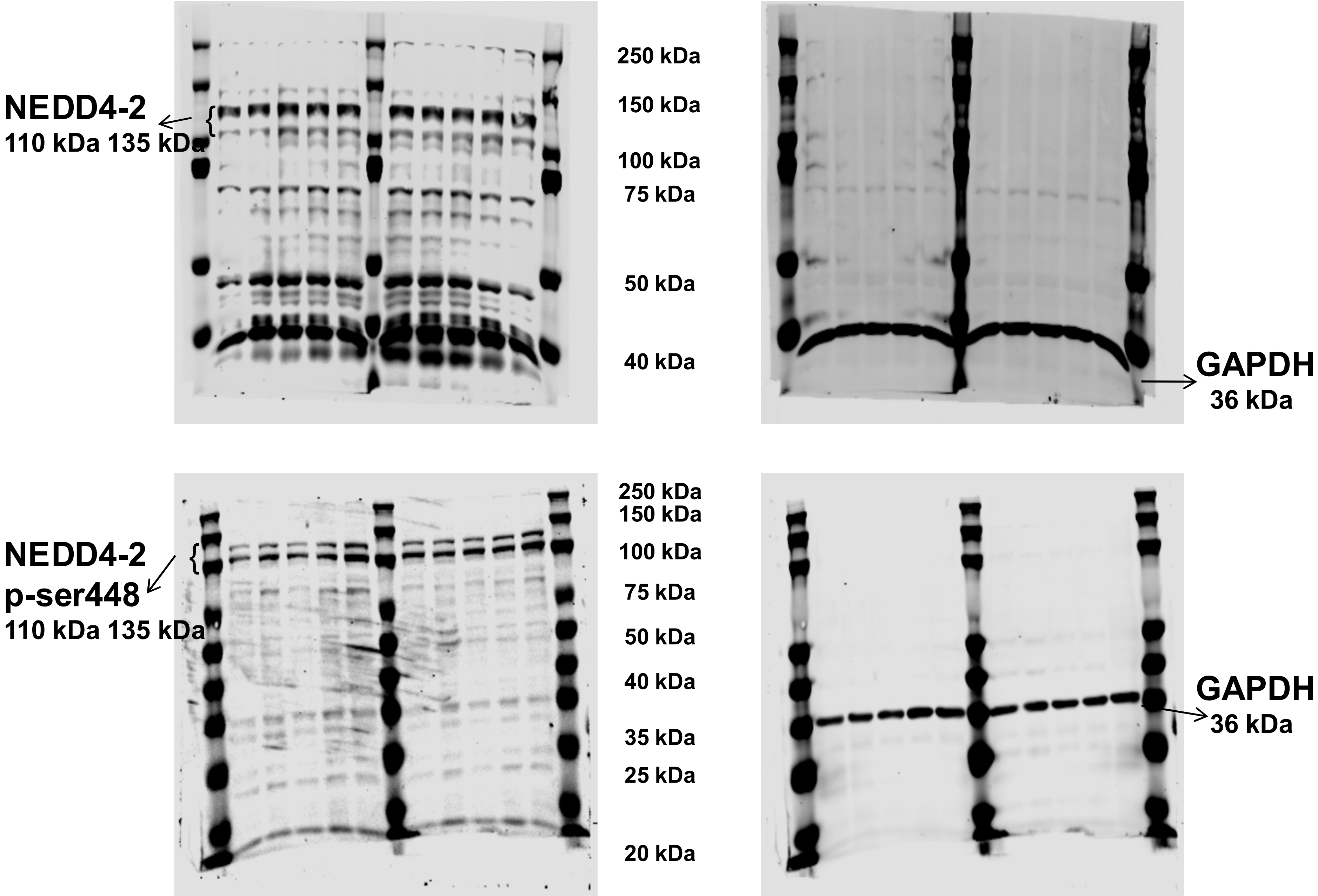
NEDD4-2
110 kDa 135 kDa
250 kDa
150 kDa
100 kDa
75 kDa
50 kDa
40 kDa
GAPDH
36 kDa
NEDD4-2
p-ser448
110 kDa 135 kDa
250 kDa
150 kDa
100 kDa
75 kDa
50 kDa
40 kDa
35 kDa
25 kDa
20 kDa
GAPDH
36 kDa

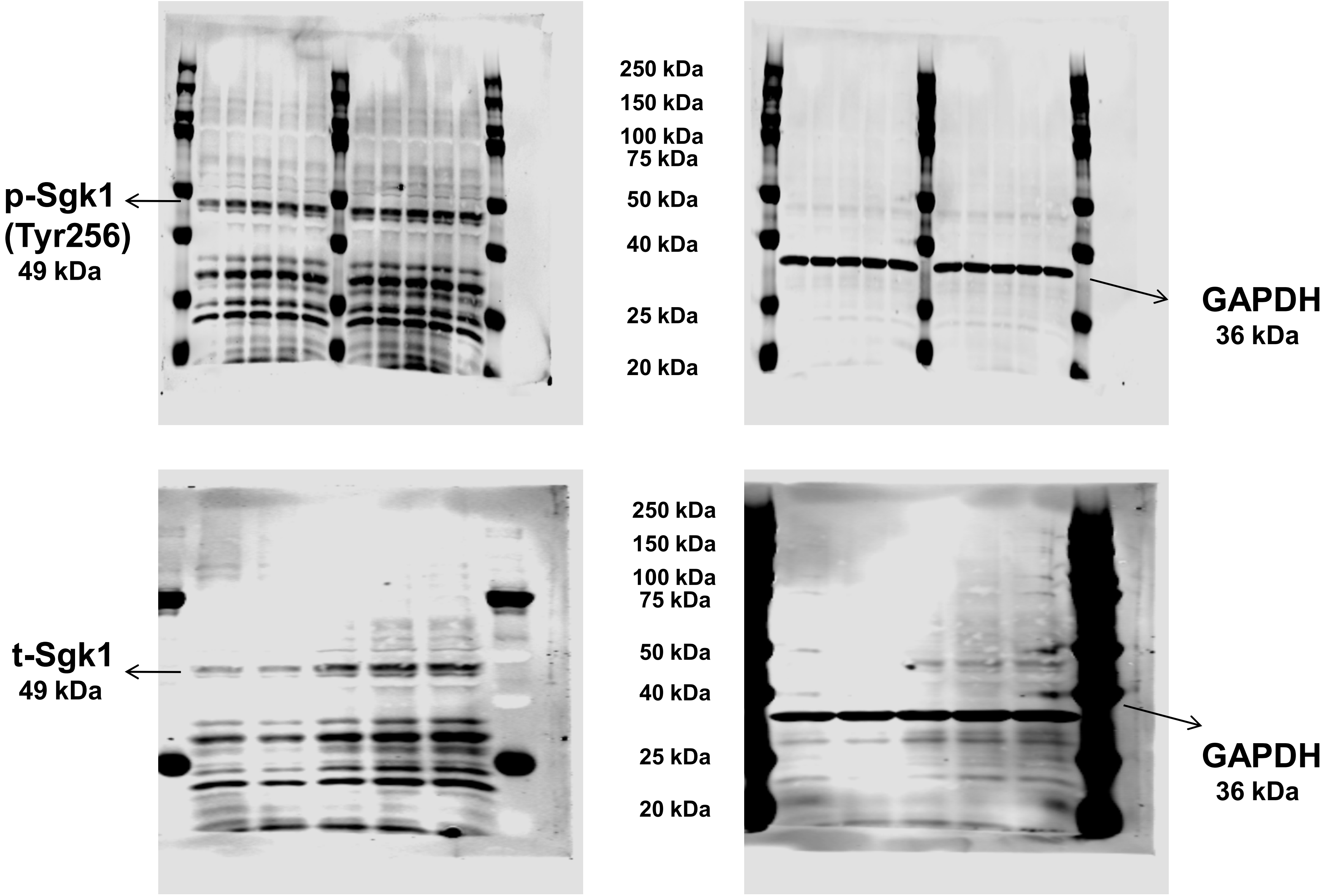
p-Sgk1
(Tyr256)
49 kDa
250 kDa
150 kDa
100 kDa
75 kDa
50 kDa
40 kDa
25 kDa
20 kDa
GAPDH
36 kDa
t-Sgk1
49 kDa
250 kDa
150 kDa
100 kDa
75 kDa
50 kDa
40 kDa
25 kDa
20 kDa
GAPDH
36 kDa
